# Age-Dependent Modulation of Cortical Transcriptomes in Spinal Cord Injury and Repair

**DOI:** 10.1371/journal.pone.0049812

**Published:** 2012-12-07

**Authors:** Anne Jaerve, Fabian Kruse, Katharina Malik, Hans-Peter Hartung, Hans Werner Müller

**Affiliations:** Molecular Neurobiology Laboratory, Department of Neurology, Heinrich-Heine-University Medical Faculty, Düsseldorf, Germany; Hertie Institute for Clinical Brain Research, University of Tuebingen, Germany

## Abstract

Both injury and aging of the central nervous system reportedly produce profound changes in gene expression. Therefore, aging may interfere with the success of therapeutic interventions which were tailored for young patients. Using genome-scale transcriptional profiling, we identified distinct age-dependent expression profiles in rat sensorimotor cortex during acute, subacute and chronic phases of spinal cord injury (SCI). Aging affects the cortical transcriptomes triggered by transection of the corticospinal tract as there was only a small overlap between the significantly lesion-regulated genes in both age groups. Over-representation analysis of the lesion-regulated genes revealed that, in addition to biological processes in common, such as lipid metabolism, others, such as activation of complement cascade, were specific for aged animals. When a recently developed treatment to suppress fibrotic scarring (anti-scarring treatment AST) was applied to the injured spinal cord of aged (22 months) and young (2 months) rats, we found that the cortical gene expression in old rats was modulated to resemble regeneration-associated profiles of young animals including the up-regulation of known repair promoting growth and transcription factors at 35 dpo. In combination with recent immunohistochemical findings demonstrating regenerative axon growth upon AST in aged animals, the present investigation on the level of gene expression strongly supports the feasibility of a successful AST therapy in elderly patients.

## Introduction

There is a growing incidence of spinal cord injury (SCI) among older individuals. The percentage of patients older than 60 years at the time of injury has increased from 4% to 11% since 2000, and the average age has increased from 28.7 years in the 1970's to the current age of 40.7 years [1]. Considering the recent and future dramatic increases in the aging population, there is substantial clinical interest in developing SCI therapies that are effective, regardless of age.

Aging has a profound effect on gene expression [2], whereby down-regulation of mitochondrial genes and up-regulation of the genes involved in inflammation mediate the conserved hallmarks of aging [2], [3]. Dysfunction of energy metabolism and increased inflammation are only two of the important factors that may render an aged nervous system more vulnerable to injury and/or diminish the efficacy of therapies originally established for the young. Transcriptional profiles of SCI in aged animals have, thus far, not been defined. Following stroke, distinct gene expression profiles in aged and young animals have been reported, which include growth-inhibitory molecules that are induced acutely and growth-promoting factors that have a delayed expression profile in the aged peri-infarcted cortex [4]. Moreover, genome-wide expression analysis of aged and young animals has revealed that different transcriptomes are responsible for stroke-induced sprouting of cortical neurons [5]. Nonetheless, selected genes relating to the regenerative response were similarly induced in both 3- and 20-months-old rats after stroke, indicating that the potential for regenerative responses in the brain remains intact at an older age [6].

SCI elicits massive changes in gene expression in the spinal cord [7] and, as we have previously reported [8], [9], in sensorimotor cortex, starting as early as 1 day post-operation (dpo). These responses increase over time. Moreover, we previously identified a regeneration-associated transcriptomic program underlying long distance axon regeneration [8], [9] along with partial functional recovery in young adult rats following local application of an anti-scarring treatment (AST) comprised of an iron chelator (2,2′-dipyridine-5,5′-dicarboxylic acid) and 8-bromo-cyclic adenosine monophosphate (8Br-cAMP) [10], [11].

In this study, we investigated the extent and nature of the difference between the dynamic cortical gene expression profiles of aged (22-months-old) and young (2-months-old) rats following thoracic corticospinal tract (CST) transection, and whether the AST-induced regeneration program can be activated in aged animals. GeneChip analyses were performed on layers V/VI of the rat sensorimotor cortex at 1, 7 and 35 dpo (days post-operation), which represented acute, subacute and chronic stages of SCI, respectively.

## Materials and Methods

### Ethics Statement

All animal experiments were conducted in agreement with national and international guidelines for animal safety and comfort. All of the surgical interventions and pre- and post-surgical animal care were provided in compliance with the German Animal Protection law and approved by the Animal Study and Ethics Committee of the State Office, Environmental and Consumer Protection of Northrhein Westfalia, LANUV NRW (Az: 8.87-50.10.34.09.081). All surgery was performed under isoflurane anesthesia, and all efforts were made to minimize suffering.

### Animal groups and experimental SCI

**Figure 1:**
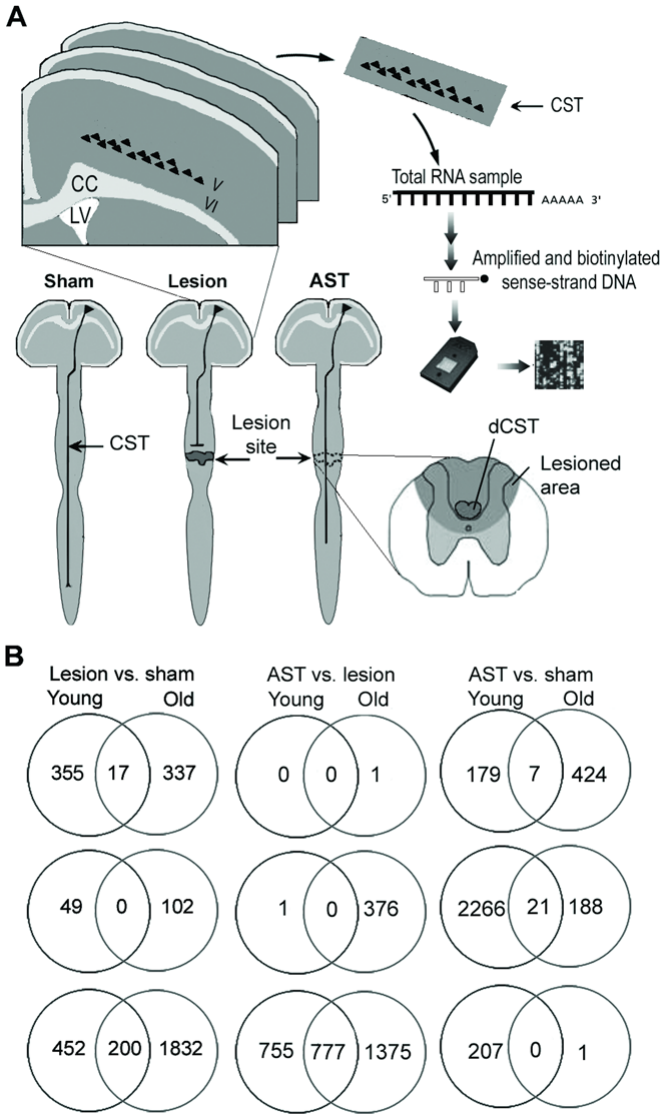
Schematic illustration of experiments. (**A**) Sensorimotor cortex layer V containing the primary motor neurons projecting their axons into the corticospinal tract (CST) and the subjacent part of layer VI were dissected out of coronal brain cryosections of sham-operated, spinal injured and AST-treated spinal injured 2- and 22-months-old rats at 1, 7 and 35 dpo (n = 4 per group). Samples from individual animals were prepared and hybridized each to an individual GeneChip. LV, lateral ventricle; CC, corpus callosum; dCST, dorsal CST. (**B**) Venn diagrams to indicate the overlap between significantly regulated genes in the groups “lesion vs. sham”, “AST vs. lesion” and “AST vs. sham” of old and young animals at 1dpo (top), 7 dpo (middle), 35 dpo (bottom). SY = sham young, AY = AST young, LY = lesioned young, SO = sham old, LO = lesioned old, AO = AST old.

Spinal cord surgery was performed under isoflurane inhalation anaesthesia (Forene, Abbott, Germany; 2–3% in O_2_ and N_2_O at a ratio of 1∶2). Dorsal spinal cord hemisection, which included transection of the corticospinal tract with a Scouten wire knife at thoracic level eight [10] (lesion group), lesion plus the anti-scarring treatment (AST group) and laminectomy alone (sham group) were investigated in 36 young (150–220 g; 2-months-old) and 36 geriatric (252–449 g; 22-months-old) female Wistar rats (HanTac:WH; Taconic, Ry, Denmark) at 1, 7 and 35 days post-operation (dpo) (Fig. 1A). In total, 72 animals in 18 groups (4 animals/group) were individually analyzed. Anti-scarring treatment (AST) consisted of 8×0.2 µl injections of the iron chelator 2,2′-bipyridine-5,5′-dicarboxylic acid (40 mM in Tris buffer) and 4×0.25 µl of 8-Br-cAMP (100 µg in Tris buffer) into the lesion site immediately after hemisection and dura suture [12]. Control lesion animals received buffer injections only. Post-operative care included heating to maintain body temperature, treatment with the pain killer Rimadyl (Carprofen, 5 mg/kg s.c.), which was delivered 3 days post injury at one injection per day, and with antibiotics (Baytril; Bayer HealthCare, Leverkusen, Germany) 0.1 ml daily orally for 1 week to avoid infections. The bladder was emptied manually.

### RNA isolation, processing and hybridization to Affymetrix GeneChips

At 1, 7 and 35 dpo, animals were deeply anesthetized (Narcoren, 100 mg/kg i.p.) and decapitated. Snap frozen left hemispheres were cut (Bregma 0.96 to −1.96) into forty 50-µm-thick coronal cryosections, which were mounted on Superfrost microscopy slides in the cryostat cabinet. Cortical layers V/VI were dissected as previously described [6]. Briefly, the appropriate pyramidal cell layer V of the sensorimotor cortex was determined by retrograde labeling of the corticospinal tract with DiI. Dissection of layer V and the closely connected layer VI was performed using head loupes, whereby the corpus callosum was used as a land mark. After dissection the cortical areas were re-inspected under the light microscope. For RNA preparation, the cortical layer V/VI was rapidly dissected out and immediately frozen on dry ice (Fig. 1A). RNA was extracted using a Qiagen RNeasy Microarray tissue kit according to the manufacturer's protocol (version November 2009) in conjunction with on-column DNase digestion. From 150 ng of total RNA, the sense-strand DNA targets were amplified and biotinylated using the two-cycle target labeling protocol (Affymetrix, Santa Clara, CA; Whole transcript sense target labeling manual 701880 Rev. 5). Samples from single animals were hybridized individually to the Rat Gene 1.0 ST array (Affymetrix) by the Core Laboratories of the Biomedical Research Center (BMFZ) at the University of Düsseldorf, Germany. Confirmation of the intactness of the RNA (RNA Integrity Numbers ranged from 8.8 to 10) and the completeness of fragmentation of the biotinylated sense strand DNA was performed with an Agilent Bioanalyzer 2100. The efficiency of hybridization and cRNA amplification were excellent and confirmed using the manufacturer's controls.

### Microarray analysis

Quantile normalization, summarization with the RMA16 algorithm and baseline transformation of the data to medians of all samples, among other analyses, were performed with GeneSpring GX software (Agilent Technologies, Santa Clara CA, USA). No arrays failed the quality-control analysis, and the correlation coefficients between biological replicate arrays were high; the lowest value was 0.95. Clustering analysis included building a global cluster of all 27,342 genes using self-organizing maps (SOM) and condition trees using average-linkage hierarchical clustering.

The total dataset was analyzed with a 3-way ANOVA (p-value cut-off 0.05, Benjamini-Hochberg correction for the false discovery rate). Comparisons between differentially expressed lesion vs. sham and between AST vs. lesion genes in young and aged animals were made by filtering for the log_2_-transformed fold-change of +/−0.25 and by performing t-tests (alpha <0.12) with the Benjamini-Hochberg correction. These settings delivered a sufficiently high number of significantly regulated genes for further evaluation using pathway analysis tools. Classification according to gene ontology was performed using the online tool DAVID [13], pathway analysis using Ingenuity Pathway Analysis program (Ingenuity Systems, Redwood City, CA, USA) and gene set enrichment analysis using ErmineJ software [14]. Microarray data have been deposited in the ArrayExpress at EMBL-EBI (Accession nr: E-MTAB-794).

### Quantitative real-time RT-PCR

Quantitative real-time RT-PCR (qRT-PCR) was used to measure the expression levels of klf7, intb7, c1qb and hbb mRNA in young animals at 35 dpo. Amplification primers for qRT-PCR analysis of the transcripts of were designed by using PrimerExpress2.0 Software (AppliedBiosystems, FosterCity, CA, USA) and subsequently tested for efficiency and specificity. The primer sequences were: Klf7, 5′-gtccgagaggcttgcataactt-3′, 3′-agaatgccaacgtatacacatcgt-5′; Itgb7, 5′-agtgccctccaagcttaacca-3′, 3′- acagtccgtgggaagtcgata-5′; Hbb, 5′-catggcaagaaggtgataaacg-3′, 3′-tcacttgaggtgacactgttcgac-5′; C1qb, 5′-ttctcaccttctgcgactatgc-3′, 3′- agaacttcgacctcgtccttc-5′. qRT-PCR was performed using SYBR green chemistry (AppliedBiosystems) and relative changes in gene expression were determined using the ΔΔCt method. Ornithine decarboxylase1 (odc1) served as reference gene. Expression levels for each sample were normalized to the corresponding sham young control group, and were calculated relative to reference gene expression.

## Results

### Differential cortical gene expression in old and young rats following SCI

When the gene expression profiles in cortical layer V/VI of both young (2 months old) and aged (22 months old) groups of spinal injured rats were compared to the respective sham animals (Fig. 1B), we found (i) that in old animals a considerable higher number of genes (2488) were significantly regulated after SCI than in young rats (753). And (ii) in both animal groups the highest number of regulated genes was observed at 35 dpi (chronic stage), whereas the least expression changes occurred at 7 dpi (subacute stage). Very surprising, there is remarkable little overlap of only approx. 7% (217 out of 3024 regulated genes) between significantly lesion- vs. sham-regulated genes in old and young animals (Fig. 1B), and at 7 dpi no significantly regulated genes overlapping in young and aged animals could be detected. Since gene expression changes in sham-operated animals were very dynamic, a finding that has also been reported by others [7], it was necessary to correlate injury responses with their respective time-matched sham profiles. Besides changes in expression of neuronal genes we detected changes in non-neuronal (glial) genes. For example, GFAP was increasingly up-regulated following SCI with higher expression in aged than in young animals.

### Biological processes regulated by SCI in old and young animals

**Figure 2:**
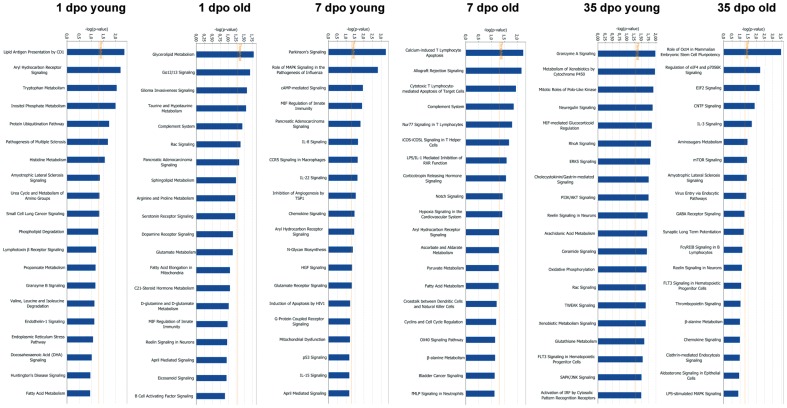
Pathway analysis of the significantly lesion- vs. sham-regulated genes in aged and young animals. Pathway analysis at different time points was done using the Ingenuity curated database of gene interactions of over 23,900 human, rat, and mouse genes. In this analysis, genes were tested for significant association in specific cell functional or signaling pathways versus random chance association in a total of gene interactions using right-tailed Fisher's exact test (Ingenuity Systems). Significance was assessed by testing the number of genes that were regulated by sprouting neurons in a specific pathway versus total number of genes in this database for that pathway (blue columns). The red line in the left graph indicates the threshold for a significant association, the −log (0.05). For example, if the pathway has a *P-score* of 10, the odds of this pathway being generated at random are less than 1 out of 10^10^.

Further, we analyzed the biological processes exerted by significantly lesion-regulated genes in aged and young animals using three different tools: over-representation analysis of biological processes by DAVID and Ingenuity Pathway analysis (IPA) and gene set enrichment analysis by ErmineJ with receiver-operator curves (ROC) and correlation resampling (COR) methods. COR method does not require a list of significantly regulated genes but analyzes expression of all genes to rank the representative biological processes. ROC takes into account in which order the genes appear in the significance list (without cut-off). Despite of the small overlap among the significant lesion-regulated genes in aged and young animals (Fig. 1B), the analysis with DAVID and ErmineJ revealed several biological processes, e.g., lipid metabolism and glycolysis that are common in both age groups (Table 1). Besides common biological processes numerous other pathways were significantly over-represented in either aged or young animals only (Table 1). On the other hand, IPA, a large curated databank analysis, revealed that different canonical cell signaling pathways were over-represented among lesion-regulated genes in young and aged animals, respectively. Similarly to DAVID and ErmineJ, the complement system was found to be increased in aged animals at 1 dpo, whereas ubiquitination pathways were up-regulated in the young animals. In addition, cAMP and chemokine signaling were predominantly up-regulated in the young animals at 7 dpo, while the complement system and Notch signaling were up-regulated in the aged animals at this subacute stage. At the chronic stage RhoA, Erk5 and PI3K/Akt were up-regulated in young animals, whereas CNTF and Oct-4 were up-regulated in aged animals at 35 dpo (Fig. 2). Together the three analysis methods with their specific features complemented each other and allowed the identification of age-specific processes in SCI.

**Table 1:** Enriched biological processes of significantly lesion-regulated genes in young and aged animals.

	1 dpo	7 dpo	35 dpo
**Young/old**	Lipid metabolism (Y:D,Cor; A:D)	Ras pathway (Y:Roc, Cor; A:Cor)	Transcription (D)
	Glycolysis (Y:Roc; A:Cor)	RNA splicing (Cor)	Translation (Y:Roc, D; A:D)
	Ras pathway (Cor)	Proteolysis (Y:Roc, Cor; A:Cor)	Ribosome biogenesis (D)
	Microtubule organization (Cor)	Chromatin modification (Cor)	RNA processing (D)
	Neurotransmitter levels (Cor)	Glycolysis (Cor)	mRNA splicing (D)
**Young**	Mitochondrial respiration (Cor)	Cholsesterol metabolism (Roc, Cor)	
	Protein degradation (Roc, Cor)	Synaptogenesis (Cor)	
	Dephosphorylation (Roc)	Dopamine transport (Roc, Cor),	
	GABA signaling pathway (Cor)	Regulation of translation (Cor)	
	Transcription initiation (Cor)	Nuclear import (Cor)	
**Old**	Potein kinase cascade (D)	Complement activation (D)	Transport (D)
	Bone development (D)	Acute inflammatory response (D)	Neuritogenesis (D)
	Apoptosis (D)	Response to wounding (D)	Neurogenesis (Roc,D)
	Chromatin modification (Cor)	Microtubule process (Roc, Cor, D)	Cell cycle (D)
	Cell adhesion (Cor)	RNA localization (Cor)	Chromatin modification (D)
	Protein processing (Cor)		MAPKKK cascade (D)
			I-κBk/NF-κB cascade (D)
			Small GTPase signaling (D)
			Protein modification (D)
			Cell-cell adhesion (Roc)

Enriched biological processes of significantly lesion-regulated genes in young and aged animals during the acute, subacute and chronic stages of SCI determined by DAVID (D), ErmineJ (Roc, receiver-operator curves, Cor, correlation resampling). In contrast to DAVID, which analyses only significantly regulated genes that have passed a defined cut-off, ErmineJ considers the entire dataset with the advantage not to be biased by cut-offs.

### Aging changes the cortical expression of genes that are relevant to SCI

**Figure 3:**
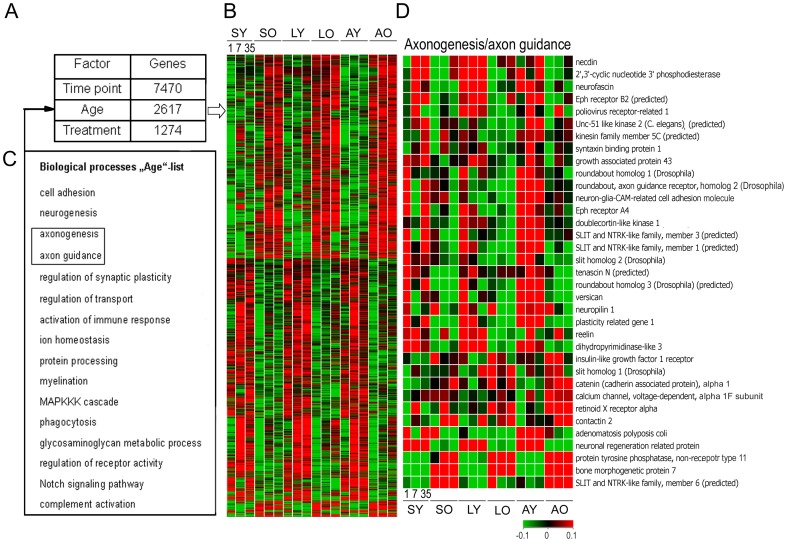
Aging changes the cortical expression of genes that are relevant to SCI. (**A**) Variance analysis of the total data set indicated that, in total, 11,361 out of 21,798 genes were significantly regulated either by time point, age or treatment. Among them 2,617 genes were regulated by age when the data were pooled over time points and treatment conditions. (**B**) Expression patterns of the age-specific genes revealed that these genes are constantly and conversely either up- or down-regulated at old *vs* young age. (**C**) Biological processes such as neurogenesis, cell adhesion and axon function, besides others, were over-represented among these age-specific genes (**D**) Genes representing the axonogenesis/axon guidance group are depicted with the respective expression profiles.

Multifactorial analysis (3-way ANOVA, p<0.05) revealed that depending on the time point, age or treatment 7470, 2617 or 1274 genes from a total of 21,798 probe sets were significantly regulated, respectively (Fig. 3). The major temporal regulation is not surprising and indicates that acute, subacute and chronic stages of SCI are distinct stages with characteristic changes in gene expression.

A substantial number of genes (2,617 from a total of 21,798 probe sets) were differentially expressed in an age-specific manner when the genes showing treatment- and time point-specific regulation were pooled. We wanted to know, which biological processes may regulate age-specific genes. Biological processes, such as axonogenesis/axon guidance, myelination, synaptic transmission, cell adhesion and activation of immune responses, were enriched in this age-specific gene group. Although not regulated by injury, these functional gene groups are very interesting in the context of SCI as they might render the aged CNS more vulnerable to injury. For example, the expression of necdin, neurofascin and plasticity related gene 1 was lower in aged animals, whereas the expression of bone morphogenic factor 7 and protein tyrosine phosphatase non-receptor type 11 was upregulated. Importantly, ANOVA analysis also revealed that there was no interrelation between the age and treatment effects, thereby demonstrating the absence of a converse regulation in aged and young animals during different treatment conditions.

### Clustering analysis

**Figure 4:**
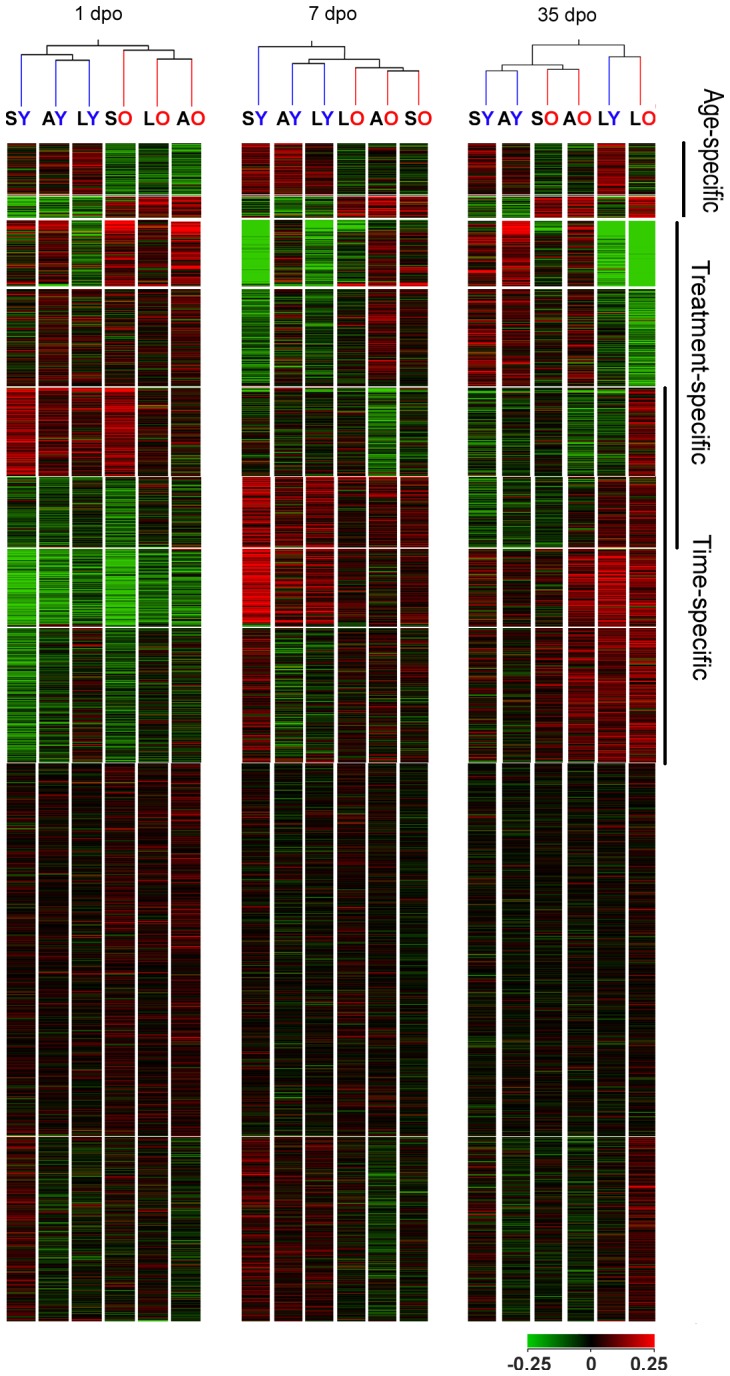
Global cluster of all 27,342 genes. Clustering of genes across all samples using the self-organizing maps. We assigned the following labels to the emerged clusters (i) genes whose expression was consistently different between aged and young animals (age-specific) and (ii) genes whose expression changed over time (time-specific) as well as (iii) genes whose expression differed between distinct treatment conditions (treatment-specific). Condition trees (average linkage hierarchical clustering of conditions at different time points) demonstrated that treatment-specific changes override the age-specific gene expression at 35 dpo (right panel, up and down, respectively). SY = sham young, AY = AST young, LY = lesioned young, SO = sham old, LO = lesioned old, AO = AST old.

Condition trees displaying the relationships between groups of animals were built at each time point because, as appeared from multifactorial analysis, the genes have very strong temporal regulation. . These trees (Fig. 4) demonstrated that at 1 dpo and 7 dpo sham, lesioned and leasioned/treated groups of old animals bore a closer similarity to each other than to the young groups. This age-specific but, to our surprise, not treatment condition-specific clustering, which we had expected, indicates how prominent the influence of aging alone is on gene expression compared to the lesion response in SCI. In contrast to the age-specific clustering of experimental groups at the acute and subacute stages of SCI, the sham and AST-treated animal groups showed close similarities at 35 dpo and could be distinguished from the lesioned animals. Unsupervised hierarchical clustering of all 72 chips showed similar trends, although the groups in clusters were not complete (Fig. S1). Clustering analysis of all 27,342 probe sets visualizes the presence of different groups such as the time-, treatment- or age-specifically regulated genes. The majority of genes remained unregulated over time and conditions, as might be expected (Fig. 4). Thus, it appears that the anti-scarring treatment elicited similar gene expression profiles regardless of age at the chronic stage of SCI.

### Synchronized cortical gene expression profiles in chronic SCI

**Figure 5:**
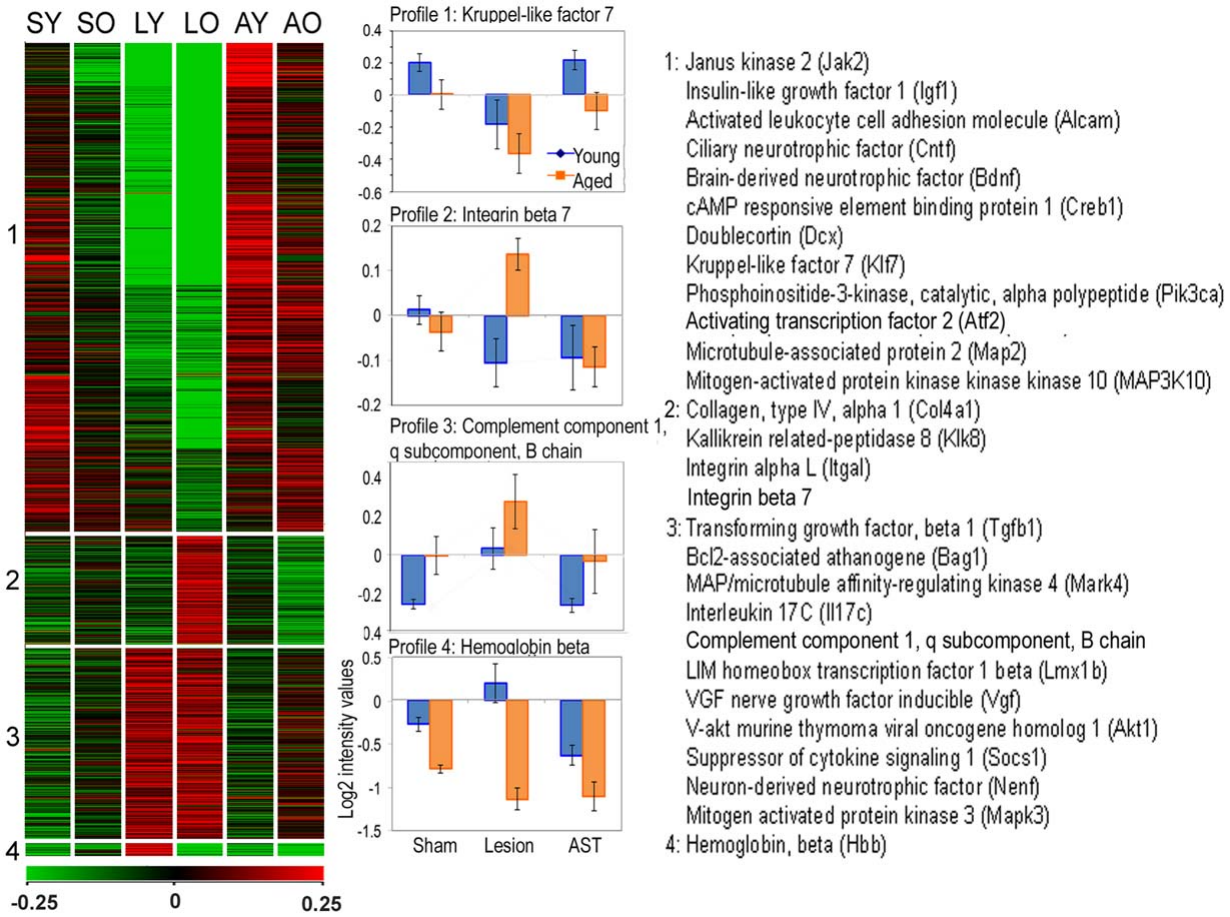
Synchronized gene expression profiles at 35 dpo. A total of 2,747 genes that were significantly affected by lesion (sham *vs.* lesion) and AST (lesion *vs.* AST) at 35 dpo is represented in the left panel. According to their expression profiles these genes could be grouped into 4 clusters with self-organizing maps (middle panel) as shown for the expression of some representative genes (right panel). SY = sham young, AY = AST young, LY = lesioned young, SO = sham old, LO = lesioned old, AO = AST old.

As we found significant effects of the anti-scarring treatment at 35 dpo, we further analysed this time point to identify distinct gene regulation profiles. In the most prominent cluster of co-regulated genes (Fig. 5, cluster 1) the AST uniformly counteracted the lesion-induced down-regulation in both aged and young animal groups back towards or even above sham expression levels. This gene cluster included growth promoting factors, such as insulin-like growth factor, ciliary neurotrophic factor and brain-derived neurotrophic factor, and signaling molecules, such as CREB1 and JAK2. The remaining three profiles (clusters 2–4) included lesion-induced up-regulation of gene expression in aged, in young or in both young and aged animals. It should be noted that AST-specific regulation neither boosted lesion-elicited responses nor did it show opposing gene regulations between young and aged animals (e.g. up-regulation by AST in young and down-regulation by AST in aged). To validate microarray profiles we measured the expression of representative genes (Fig. 5) using quantitative qRT-PCR. It turned out that the expression patterns were in general agreement with the microarray data (Table 2).

**Table 2:** Validation of the expression of selected transcripts by qRT-PCR.

		Microarray					qRT-PCR				
		Fold changes			p-values		Fold changes			p-values	
Gene	EntrezID	Sham	Lesion	AST	Sh vs L	L vs A	Sham	Lesion	AST	Sh vs L	L vs A
Klf7	363243	1.00±0.05	0.78±0.07	1.02±0.05	*	*	1.00±0.09	0.69±0.07	1.07±0.07	***	n.s.
Intb7	25713	1.00±0.03	0.92±0.03	0.93±0.05	*	n.s.	1.00±0.21	0.89±0.19	0.57±0.16	n.s.	**
C1qb	29687	1.00±0.04	1.24±0.06	1.00±0.03	**	**	1.00±0.11	1.18±0.13	1.08±0.11	*	n.s.
Hbb	24440	1.00±0.06	1.33±0.17	0.83±0.06	n.s.	*	1.00±0.08	1.11±0.18	0.85±0.04	n.s.	**

Validation of changes in mRNA expression by quantitative real-time RT-PCR (qRT-PCR) for genes representative of expression patterns of young animals at 35 dpo identified by the GeneChip analysis (Fig. 5). mRNA expression levels (mean fold change ± standard error, n = 3–4 animals) relative to young sham animals determined with microarray and by qRT-PCR. Microarray values are given as ratios relative to normalization and RMA levels derived from Genespring. qRT-PCR values are ratios relative to ornithine decarboxylase 1 (ODC, houskeeping gene) expression. For all genes measured, qRT-PCR corroborated the rank order of magnitude of expression observed with microarrays. Abbrevations: Kruppel-like factor 7 (Klf7), integrin beta 7 (Intb7), complement component 1 q subcomponent, B chain (C1qb), hemoglobin beta (Hbb), Sh, sham; ls, lesion.

* p<0.05,

** p<0.01,

*** p<0.001,

n.s., not significant (student's *t*-test).

## Discussion

Using a systematic microarray approach, we demonstrate that the local anti-scarring treatment to the injured spinal cord in aged and young animals appears to elicit a similar gene expression profile at the chronic stage of traumatic SCI.

The cortical transcriptomes following SCI were initially largely age-specific, because there was little overlap of significantly SCI-regulated genes between aged and young animals. However, on the pathway level, there were common lesion-induced age-independent gene regulations found in processes such as glycolysis, RNA splicing and proteolysis. On the other hand, several of the biological processes exerted by regulated genes were predominant either in young or aged animals. For example, complement activation was upregulated in aged animals. This particular pathway might be especially unfavorable for regeneration, as it has been shown that the inhibition of complement pathways promotes functional recovery and reduces tissue damage [15]–[17]. Complement component 3, which is generated upon activation of the complement pathway, leads to production of C3b and iC3b. The latter complement components are opsonins that target apoptotic cells and promote their clearance by macrophages and microglia [18]. We found that their levels were higher in aged compared to young lesioned animals in all three time points. On the other hand, C3a has recently been shown to inhibit pro-inflammatory cytokines and to contribute to stem cell chemotaxis into areas of inflammation, potentially enhancing tissue repair [19]. Complement component 1 q subcomponent beta polypeptide (C1qB) was upregulated at 1 dpo and 35 dpo, whereby the expression levels were significantly higher in aged animals than in young rats at 1 dpo. In fact, upregulation of this recognition component of the complement system C1q may be a general response to injury as it is also upregulated in brain ischemia/reperfusion [20]. Cortical deafferentation has been shown to cause elevations in striatal C1qB mRNA that coincided temporally and overlapped anatomically with the course of degeneration of corticostriatal afferent fibers [21]. In the absence of other complement proteins such as C3a or C5a (this is, at least, the case for the respective transcripts in young animals at 1 dpo in the present investigation), C1q improves neuronal viability and neurite outgrowth [22]. By analogy with the known role of complement factors in fat tissue it is proposed, that local expression of these factors may play a role in the regulation of fatty acid homeostasis and in energy metabolism cross-talk between different compartments of the peripheral nerve [23].

Lesion-regulated genes were enriched for lipid metabolism, which in the context of SCI has thus far only received little attention. Fatty acids play an important role in neurite outgrowth as structural building blocks for extensive membrane biosynthesis. Polyunsaturated fatty acids (PUFA) such as arachidonic acid (AA) and docosahexaenoic acid (DHA), which are taken up by neurons, provide the necessary flexibility and fluidity for membranes [24]. Acyl-CoA synthetases (ACSs) are rate-limiting for fatty acid internalization and, in line with that, ACS2 enhances neurite outgrowth by promoting PUFA internalization [25]. Acyl-CoA synthetase long-chain family member 1 is upregulated at 1 dpo in young animals.

According to ErmineJ and IPA analyses proteolysis was one of the significant biological processes regulated in young animals at 1 dpo. Cathepsins, cysteine proteases, are a good example of this group of potent proteases that degrade intracellular proteins engulfed by lysosomes, but also extracellular elements such as elastin, fibronectin, laminin and collagens. Cathepsin S was upregulated in young lesioned animals, whereas cathepsin K transcripts were enhanced in aged animals at 1 dpo. Cathepsin S was proposed to play a role in the migration and activation of microglia to protect facial motoneurons from axotomy-induced injury [26]. The mRNA and protein of cathepsin K, which is known for its role in osteoclast-mediated bone resorption, were recently detected in neuronal and non-.neuronal cells of the mouse brain [27]. It was proposed that cathepsin K coordinates the proteolytic network in the brain to maintain CNS homeostasis. Of note, by screening the GENSAT gene expression atlas [28], we found a surprisingly strong cathepsin K signal localized in the dorsal corticospinal tract in EGFP-Cathepsin K mice (http://www.gensat.org/imagenavigator.jsp?imageID=13651) suggesting a function of this cathepsin in SCI that needs further investigation.

Mitochondria-associated genes which are involved in energy production were enriched in lesioned animals indicating the importance of energy supply for cortical motor neurons (motor cortex layer V) after axotomy. These cells have a higher energy demand than the rest of the cortex [29]. Most genes involved in glycolysis were upregulated already at 1 dpo.

It is interesting to note, that at 7 dpo no overlap of regulated genes could be observed between young and aged rats in cortical layer V/VI after SCI (Fig. 1B). Genes commonly induced after neural injury like, e.g., activating transcription factor 3 (ATF3) were, however, regulated in both young and aged animals but regulation did not reach significance at 7 dpo. Age-specific expression profiles have also been reported in stroke [4] and stroke-induced sprouting [5]. Paradoxically, in those studies the sprouting neurons in aged animals showed up-regulation of genes encoding axon growth-inhibitory myelin proteins and ephrin receptors. Therefore, the whole transcriptome matters, although it might include genes which seem unfavourable to regenerative growth. In our study there was no significant reciprocal gene regulation in the two age groups. Lesion-dependent gene regulation ranked behind aging-dependent regulation at the acute and subacute stages of SCI. This is due to the rather high proportion of age-specific regulated genes (9.5% of total probe sets), which is in agreement with the literature [2], [3]. Moreover, the age-specific regulated genes were preferentially involved in axonal functions, which is highly relevant to SCI. Thus we speculate that the altered expression of these genes in aged animals might render the aged CNS more vulnerable to injury. For example, necdin, expressed predominantly in postmitotic neurons interacting with neurotrophin receptors, was expressed at lower levels in aged animals. Deficiency of necdin has been shown to impinge axonal outgrowth [30] leading to an increased susceptibility of motoneurons to neurotrophic factor deprivation and is associated with impairment of motor function [31]. Necdin downregulates p53 acetylation levels by forming a stable complex with p53 and Sirt1 to protect neurons from DNA damage-induced apoptosis. Moreover, transfection with necdin accelerated neurite outgrowth of cortical neurons [32].

Neurofascin, a cell surface protein which belongs to the immunoglobulin superfamily, was expressed at lower levels in aged animals than in the young. This protein has been shown to regulate mechanisms of plasticity including neurite outgrowth, the formation of postsynaptic components and the stabilization of neural structures [33].

Plasticity related gene 1 (Prg1), neuron-specific membrane-associated lipid phosphate phosphatase, was expressed at lower levels in aged animals. Prg1 is associated with axon growth during development and regenerative sprouting following entorhinal cortex lesion as well as in synaptic plasticity and maintenance of the differentiated state throughout adulthood [34]. Although, we did not observe an induction of Prg1 after SCI, as has been shown after lesioning of the entorhinal cortex [34], lower levels of this protein in SCI, however, might be just one of the examples for lowering the regenerative potential of the aged CNS. Note, that the majority of these age-specifically regulated and neuronal expressed genes are down-regulated in aged animals thus supporting the transcriptional repression hypothesis during aging.

Some aging-specific alterations in gene expression might even be positive and/or reflect the coping of the CNS with cellular stress at higher age. For example, we found bone morphogenetic protein 7 (BMP7) expressed at higher levels in aged animals. BMP7 has neuroprotective capacity as it can enhance dendritic growth and protect cultured neurons from oxidative stress [35] as well as reduce ischemia- or neurotoxin-mediated neurodegeneration *in vivo* via anti-apoptotic mechanisms [36].

Anti-scarring treatment, which was locally applied immediately after the lesion, elicited massive changes in gene expression in the cortex at the chronic stage of SCI. As early as 1 and 7 dpo, the variation in gene expression was much less in AST-treated animals relative to lesion-only animals. At the chronic stage (35 dpo) of SCI upon AST the profiles in aged and young animals were more similar to each other than at earlier time points. The profiles included the up-regulation of many lesion-dependent down-regulated genes (e.g., growth factors) to the sham-level or higher. This finding is consistent with recent reports demonstrating that an activated regeneration program underlies the increased axonal regrowth of CST in young animals upon AST [10], [11]. As shown here, down-regulated by lesion and upregulated by AST was the following very interesting group of growth and transcription factors: ciliary neurotrophic factor (CNTF), insulin-like growth factor 1 (IGF-1), brain-derived growth factor (BDNF), Kruppel-like growth factor 7 (KLF7), doublecortin (DCX), CREB1 and activating transcription factor 2 (ATF2). CNTF is able to prevent motoneuron degeneration after axotomy [37], [38] and to support corticospinal motor neuron growth via direct mechanisms [39]. Recently, it was shown that cortical neurons up-regulate a CNTF-mediated neuroprotective signalling pathway in response to chronic insults or stress in the pathogenesis of multiple sclerosis [40]. Similarly, IGF-1 together with BDNF was shown to enhance specifically axon outgrowth of corticospinal motor neurons [41]. DCX, an endogenous marker of immature neurons, was recently hypothesized to play a role in cortical plasticity and brain repair [42]. DCX-positive cells were present in the whole primate cerebral cortex and expressed glial and/or neuronal markers. However, only the DCX/GFAP positive cells were able to proliferate and reacquire progenitor characteristics [42]. KLF7 is required for neuronal morphogenesis and axon guidance in selected regions of the nervous system including the cortex [43]. In fact, Moore et al. [44] showed that KLF7 is the most effective among KLFs to promote neurite outgrowth in cortical and retinal ganglion primary cultures and, moreover, overexpression of KLF7 engineered for transcriptional activation promotes axon regeneration in the adult CST [45]. We found ATF2 elevated in aged animals at 35 dpo upon AST treatment. Interestingly, following axotomy, ATF2 is reduced in the dorsal motor nucleus of the vagus nerve and in the hypoglossal nucleus in rats [46], and it is upregulated in sprouting cortical neurons following stroke [5]. Rapid and persistent down-regulation of ATF2 is a constituent of the long-term neuronal stress response and the reappearance of ATF2 after several weeks indicates the normalization of neuronal gene transcription following brain injury [47].

Interestingly, the cell adhesion molecule ALCAM was found upregulated in our investigation and also in the study by Li et al. [5]. ALCAM is involved in neurite extension via heterophilic and homophilic interactions [48], [49] and it may further play a role in the binding of T- and B-cells to activated leukocytes and/or mediate interactions between cells of the nervous system. Hemoglobin is usually not associated with neuronal cells. However, hemoglobin protein chains were recently shown to be expressed by neurons and astrocytes and might play a novel role in lesion responses [50]. Hemin-induced HbA and HbB expression in cultured neurons was reduced by deferoxamine treatment [51]. As our AST treatment also includes an iron chelator, the finding that lesion-induced HbB in young rats was reduced by AST is in line with previous observations.

From the present findings we conclude, that age cannot be neglected as a factor in therapeutic responses. As was shown here for SCI and as others have reported for stroke, age-related changes produce distinct transcriptomes. However, despite the initial age-specific differences in gene expression, the local therapeutic intervention in the injured spinal cord by AST activated similar molecular programs in both aged and young animals. Thus AST overruns the age-specific differences in transcriptomic lesion responses. Together with previous immunohistological observations regarding axonal regrowth upon AST in aged animals [12], the present observations on the level of gene expression are not only in line with but substantially extend the previous findings and further imply that pharmacological anti-scarring treatment offers a potential therapeutic option for elderly SCI patients.

## Supporting Information

Figure S1 **Unsupervised hierarchical clustering of all 72 chips.** The underlying colored bars point to different clusters of animals. The red bar to the left indicates the clustering of young and old sham and AST animals at 5 weeks post-operation (wpo). This cluster represents at least half of the sham and AST animals of both ages at 5 wpo (50% of SY, SO, AY and 75% of AO). The red bar on the right end represents the clustering of half of the lesioned young and old animals at 5 wpo. Moreover, the clustering results support the age-specific clustering at 1 dpo (green bars) and 7 dpo (blue bars) similar to the clustering of groups as shown in Fig. 4. Clearly, at 5 wpo the similarity between sham and AST animals of both ages is closer than between lesion and sham or lesion and AST. Although, the 5 week lesioned young and old animals form a rather small group, it clearly differs from the AST and sham animals of this time point. It appears that within 5 weeks the treatment in young and old animals elicits similar effects regardless of age. SY = sham young, AY = AST young, LY = lesioned young, SO = sham old, LO = lesioned old, AO = AST old.
(TIF)
