# Supplementary figures and images for: Age-Dependent Modulation of Cortical Transcriptomes in Spinal Cord Injury and Repair
Source: PLoS One. 2012 Dec 7;7(12):e49812. doi: 10.1371/journal.pone.0049812 (PMC3517588; doi:10.1371/journal.pone.0049812)

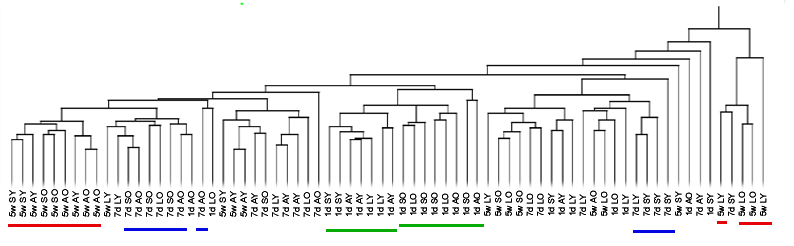

Supplement: Figure S1 — Unsupervised hierarchical clustering of all 72 chips. The underlying colored bars point to different clusters of animals. The red bar to the left indicates the clustering of young and old sham and AST animals at 5 weeks post-operation (wpo). This cluster represents at least half of the sham and AST animals of both ages at 5 wpo (50% of SY, SO, AY and 75% of AO). The red bar on the right end represents the clustering of half of the lesioned young and old animals at 5 wpo. Moreover, the clustering results support the age-specific clustering at 1 dpo (green bars) and 7 dpo (blue bars) similar to the clustering of groups as shown in Fig. 4. Clearly, at 5 wpo the similarity between sham and AST animals of both ages is closer than between lesion and sham or lesion and AST. Although, the 5 week lesioned young and old animals form a rather small group, it clearly differs from the AST and sham animals of this time point. It appears that within 5 weeks the treatment in young and old animals elicits similar effects regardless of age. SY = sham young, AY = AST young, LY = lesioned young, SO = sham old, LO = lesioned old, AO = AST old. (TIF) [file pone.0049812.s001.tif]
